# Association analysis of the dopaminergic receptor 2 gene *Tag1B* rs1079597 and personality traits among a cohort of professional athletes

**DOI:** 10.5114/biolsport.2025.139470

**Published:** 2024-08-30

**Authors:** Monika Michałowska-Sawczyn, Kinga Humińska-Lisowska, Krzysztof Chmielowiec, Jolanta Chmielowiec, Aleksandra Strońska-Pluta, Aleksandra Suchanecka, Łukasz Zadroga, Myosotis Massidda, Carla Maria Calò, Remigiusz Recław, Anna Grzywacz

**Affiliations:** 1 Faculty of Physical Education, Gdansk University of Physical Education and Sport, Poland; 2 Department of Hygiene and Epidemiology, Collegium Medicum, University of Zielona Góra, 28 Zyty St., 65-046 Zielona Góra, Poland; 3 Independent Laboratory of Behavioral Genetics and Epigenetics, Pomeranian Medical University in Szczecin, Poland; 4 Student Scientific Club of Independent Laboratory of Genetics and Behavioral Epigenetics, Pomeranian Medical University in Szczecin, Poland; 5 Department of Medical Sciences and Public Health, University of Cagliari, Italy; 6 Department of Life and Environmental Sciences, University of Cagliari, Italy

**Keywords:** Professional athletes, Sport, DRD2, Personality, NEO Five-Factor Inventory

## Abstract

Research into sports participation has increasingly pointed to inherent biological mechanisms as influential factors alongside psychosocial and environmental elements. The dopaminergic D2 receptor is a strong candidate gene for physical activity behaviour, given its role in locomotor control and reward mechanisms. Hence, this study aimed to analyse the association of the *DRD2* gene *Tag1B* rs1079597 polymorphism with personality traits in elite athletes. The study group consisted of 395 volunteers. Of these, 163 were professional athletes (22.56 ± 5.9; M = 114, F = 49), and 232 were controls (22.07 ± 4.3; M = 150, F = 82). The MINI-International Neuropsychiatric Interview and the NEO Five-Factor Inventory were administered in both groups. Genotyping was performed using the real-time PCR method. Statistical analysis was performed: genotypes and alleles frequencies were compared using the chi-square test and the relations between *DRD2 Tag1B* rs1079597 variants, professional athletes and control participants and the NEO Five-Factor Inventory were analysed with the factorial ANOVA. Statistically significant differences were found in the frequency of *DRD2 Tag1B* rs1079597 genotypes and alleles in the group of professional athletes group compared to the control group. The GG genotype and G allele were significantly more frequent in the group of professional athletes (G/G 0.79 vs G/G 0.66; A/A 0.04 vs A/A 0.03; A/G 0.17 vs A/G 0.31, *p = 0.0056*; G 0.87 vs. G 0.81; A 0.13 vs. A 0.19, *p* = 0.0281) compared to the control group. The professional athletes’ participants, compared to the controls, obtained significantly higher scores in the assessment of NEO-FFI Extraversion (*p* = 0.0369) and Conscientiousness (p < 0.0001) scales. Additionally, there was a statistically significant effect of DRD2 rs1079597 genotype association with being a professional athlete on the Openness scale (F_2.3389_ = 3.07; *p* = 0.0475; η^2^ = 0.015) and on the Conscientiousness scale (F_2.3389_ = 3.23; *p* = 0.0406; η^2^ = 0.016). This study highlights the significant associations between the *DRD2 Taq1B* polymorphic site and personality traits in a group of professional athletes. It also demonstrates the association of *Taq1B* polymorphism and professional sportsmanship with personality traits measured by NEO-FFI. The results suggest that genetic factors and professional sportsmanship both shape an athlete’s personality traits.

## INTRODUCTION

Dopamine is an essential neurotransmitter secreted by dopaminergic neurons in the central nervous system. Dopamine influences a number of brain functions, including attitudes towards making ‘risky decisions’ as the leading role of dopamine, particularly the D2 receptor isoforms, is to integrate motivation, action and emotion [1]. According to Hranilovic et al. [2], this seems the most relevant for new stimuli. Previous research has demonstrated that the role of the dopamine D2 receptor (DRD2) differs in sporting behaviour in humans and animals [3–5]. DRD2 regulates the release of dopamine, which influences motivation and rewarding behaviour [6].

The *DRD2* gene encodes the dopamine D2 receptor, which is located on chromosome 11q23.2. This gene consists of eight exons transcribed into an mRNA of 2713 kb length and then translated into a protein of 443 amino acids. The omission of the sixth exon leads to the production of a short form of the receptor, which differs from the longer form of the receptor protein, which is 29 amino acids long. The two D2 receptor isoforms differ in their affinity for inhibitory G proteins [7]. Within the *DRD2* gene, there are a number of polymorphisms known to drive individual differences in impulsivity and addiction among a group of non-athletes. Another study, conducted on a group of European women of Caucasian origin, reports a link between the effectiveness of sequential motor learning. This skill appears to be essential for athletes who perform complex sets of movements and present a high level of coordination [8]. The rs1079597 (*TaqIB*) polymorphism is located in the first intron of the *DRD2* gene. According to studies, there is an association between the C(B1) allele and a decrease in DRD2 density in the striatum [9, 10].

Dopamine secretion and metabolism show strong effects on personality traits [11]. For this reason, several researchers have found that personality dimensions are linked to dopamine-related genes [12]. Personality influences behaviour, lifestyle and maintenance of normal function throughout life. The most common factor model used to study personality is the Big Five model. As the name suggests, it consists of five traits: Conscientiousness, Openness, Extraversion, Agreeableness and Neuroticism [13–15]. These five dimensions determine differences between people and are related to emotions, behaviour, motivation and cognition [16]. Conscientiousness describes a tendency to control impulses and act in a socially acceptable way [17]. On the other hand, Openness is related to divergent thinking and intelligence. Furthermore, it has been noted that Openness is dependent on dopamine function, particularly in the prefrontal cortex [18]. Extroversion, on the other hand, is characterised by assertiveness, sociability and excitability. Individuals in whom Extraversion is the dominant trait appear to be more prevalent in social settings [19]. Agreeableness is a tendency towards compassion and cooperation. It is also described by characteristics such as trust, altruism and other pro-social behaviour [20]. Neuroticism is characterised by large mood swings and frequent experiences of feelings of anxiety, worry, anger, fear, jealousy, frustration, guilt, depressive moods and loneliness [21, 22]. The Revised NEO-FFI Personality Inventory is the most widely used questionnaire to analyse these personality traits [23].

The study had three specific objectives: (1) to conduct an association analysis of the *DRD2* gene *Tag1B* rs1079597 polymorphism in a group of professional athletes compared to controls; (2) to perform a personality trait analysis, measured by the NEO Five-Factor Inventory, and to compare it between the aforementioned groups; and (3) to conduct an interaction analysis of the measured personality traits, rs1079597 and professional sport participation.

## MATERIALS AND METHODS

### Participants

The study group consisted of 395 volunteers. Of these, 163 professional athletes (mean age = 29.44, SD = 10.74; F = 49%, M = 51%) and 232 controls (mean age = 26.91, SD = 10.10; F = 80%, M = 20%). The study was approved by the Bioethics Committee for Clinical Research of the Regional Medical Society in Szczecin, Marii Skłodowskiej-Curie 11 Street (protocol no. 13/KB/VI/2016, 8 December 2016). Before entering the study, all individuals provided written informed consent. The study was carried out at the Independent Laboratory of Health Promotion. Both professional athletes and the control group were subjected to a psychiatric evaluation, which involved the Mini International Neuropsychiatric Interview (MINI) and NEO Five-Factor Personality Inventory (NEOFFI). The study group consisted of participants in international or national competitions in various sports disciplines, including martial arts – 84% of the participants (karate, n = 30; judo, n = 33; boxing, n = 24; wrestling, n = 25; ju-jitsu, n = 25; volleyball, n = 11, handball league, n = 15). Professional sportsmen and women took part in sports competitions in the last year prior to the study and have been systematically involved in training for at least five years.

### Psychometric tests

The MINI-International Neuropsychiatric Interview is a structured diagnostic interview which assesses psychiatric diagnoses based on DSM-IV and ICD-10 criteria. The Five-Factor Inventory is composed of six components for the following five traits: Neuroticism (selfawareness, hostility, depression, impulsivity, anxiety, susceptibility to stress), Extroversion (assertiveness, activity, emotion seeking, positive emotions, warmth, sociability), Openness to experience (aesthetics, values, fantasy, actions, ideas, feelings), Agreeableness (straightforwardness, compliance, trust, altruism, modesty, tenderness), Conscientiousness (order, striving for achievements, consideration, duty, competence, self-discipline) [23]. The results of the NEO-FFI were reported as sten scores. In accordance with the Polish standards for adults, the raw scores were converted to the sten scale, which ranges from 1 to 10. This scale categorises scores as follows: 1–2 corresponds to very low results, 3–4 to low results, 5–6 to average results, 7–8 to high results, and 9–10 to very high results.

### Laboratory procedures

Vacuum blood collection kits containing EDTA anticoagulant were used to collect 9 ml of whole blood from the ulnar vein. The QIAamp ® DNA Mini Kit (QIAGEN, Hilden, Germany) was used for the purification of genomic DNA. Genotyping was conducted with the realtime PCR method using the oligonucleotide LightSNiP probes (TibMolBiol, Berlin, Germany) on the LightCycler 480II instrument (Roche Diagnostics, Basel, Switzerland). The fluorescence signal was plotted as a function of temperature to provide melting curves for each sample. The peaks were read at 57.4°C for the G allele and 62.25°C for the A allele.

### Statistical Analysis

The HWE software was utilised to assess the concordance between the distribution of allele frequencies and Hardy-Weinberg equilibrium (https://wpcalc.com/en/equilibrium-hardy-weinberg/ (Date of access 05 April 2023). The interaction between *DRD2 Tag1B* rs1079597 variants, professional athletes and control participants and the NEO Five-Factor Inventory were analysed using a multivariate analysis of factor effects ANOVA [NEO-FFI scale × genetic feature × control and professional athlete × (genetic feature × control and professional athlete)]. The condition for homogeneity of variance has been met (Levene test p > 0.05). The variables under analysis did not follow a normal distribution. The Mann-Whitney U test was used to compare the scores of the NEO Five-Factor Inventory (Neuroticism, Extraversion, Openness, Agreeableness and Conscientiousness) in the analysed groups. The genotypes and alleles frequencies of *DRD2 Tag1B* rs1079597 were compared using the chi-square test. All calculations were carried out with the STATISTICA 13 (Tibco Software Inc, Palo Alto, CA, USA) for Windows (Microsoft Corporation, Redmond, WA, USA).

## RESULTS

The alleles frequency of analysed rs1079597 accorded with Hardy-Weinberg’s equilibrium in the control participants but did not in the professional athletes’ group (Table 1.).

**TABLE 1:** Hardy-Weinberg’s equilibrium for professional athletes and controls group.

Hardy-Weinberg equilibrium including analysis for ascertainment bias		Observed (Expected)	allele freq	χ2 (*p*-value)

	*DRD2* rs1079597			
Professional athletes n = 163	G/G	128 (123.7)
	A/A	7 (2.7)	p (ins)= 0.87	8.982
	A/G	28 (36.6)	q (del)= 0.13	(0.0027)*
Control n = 232	G/G	152 (153.2)
	A/A	7 (8.2)	p (ins)= 0.81	0.2483
	A/G	73 (70.7)	q (del)= 0.19	(0.6183)

*n* – number of participants, *p* – statistical significance, χ^2^ test,

* – significant statistical differences.

Statistically significant differences were found in the frequency of *DRD2 Tag1B* rs1079597 genotypes in the tested professional athletes group compared to the control group (G/G 0.79 vs G/G 0.66; A/A 0.04 vs A/A 0.03; A/G 0.17 vs A/G 0.31, χ^2^ = 10.370, *p* = 0.0056). Additionally, significant differences in the frequency of rs1079597 alleles were found between professional athletes and the control group (G 0.87 vs. G 0.81; A 0.13 vs. A 0.19, χ^2^ = 4.820, *p* = 0.0281) (Table 2).

**TABLE 2:** Frequency of genotypes and alleles of the *DRD2* gene rs1079597 in the professional athletes and controls.

*DRD2* rs1079597	Genotypes			Alleles	

	G/G n (%)	A/A n (%)	A/G n (%)	G n (%)	A n (%)
Professional athletes n = 163	128 (78.53%)	7 (4.29%)	28 (17.18%)	284 (87.12%)	42 (12.88%)
Control n = 232	152 (65.52%)	7 (3.02%)	73 (31.47%)	377 (81.25%)	87 (18.75%)
χ^2^ (p-value)	10.370 (0.0056)*			4.820 (0.0281)*	

*n* – number of participants, *p –* statistical significance, χ^2^ test,

* – significant statistical differences.

Table 3 presents the means and standard deviations of all NEOFFI results for both professional athletes and control participants. The professional athletes’ participants, compared to the control group, obtained higher scores in the assessment of NEO-FFI Extraversion (6.84 vs. 6.37; Z = 2.086; *p* = 0.0369) and NEO-FFI Conscientiousness (7.19 vs. 5.88; Z = -5.854; *p* ≤ 0.000) scales.

**TABLE 3:** NEO Five-Factor Inventory sten scores in professional athletes and controls.

NEO Five-Factor Inventory	Professional athletes (n= 163)	Control (n = 232)	Z	(p-Value)
Neuroticism scale	4.81 ± 2.01	4.61 ± 1.90	0.659	0.5097
Extraversion scale	6.84 ± 2.01	6.37 ± 1.99	2.086	0.0369*
Openness scale	4.86 ± 2.25	4.53 ± 1.63	1.554	0.1201
Agreeability scale	5.94 ± 3.75	5.66 ± 2.07	0.417	0.6769
Conscientiousness scale	7.19 ± 2.09	5.88 ± 2.12	5.854	0.0000*

*p*, statistical significance with Mann–Whitney U-test; *n*, number of participants; M ± SD, mean ± standard deviation;

* statistically significant differences.

Table 4 summarises the results of the 2 × 3 factorial ANOVA of the NEO Five-Factor Personality Inventory (NEO-FFI) sten scales and *DRD2 Tag1B* rs1079597. A significant statistical impact of being a professional athlete and *DRD2* rs1079597 genotype was demonstrated for the score of the Openness scale. There was a statistically significant effect of *DRD2* rs1079597 genotype interaction and being a professional athlete or nor (control group) on the Openness scale (F_2.3389_ = 3.07; *p* = 0.0475; η2 = 0.015). The power observed for this factor was 60%, and approximately 1,5% was explained by the polymorphism of the *DRD2* rs1079597 and being a professional athlete or nor (control group) on Openness score variance. Table 5 presents the post hoc test results.

**TABLE 4:** The results of 2 × 3 factorial ANOVA for professional athletes and controls, NEO-FFI and DRD2 gene rs1079597.

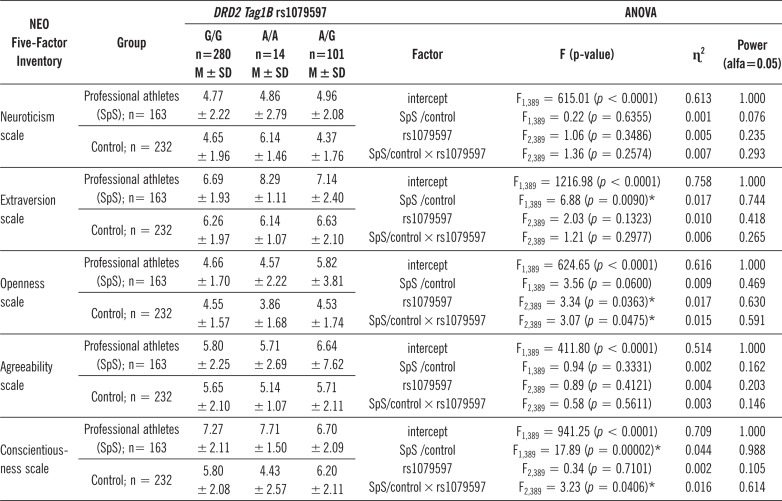

* – significant result; M ± SD – mean ± standard deviation.

**TABLE 5:** Post hoc test (Least Significant Difference) analysis of interactions between the professional athletes /Control and rs1079597and Openness scale.

rs1079597 and Openness scale							
		{1} M=4.66	{2} M=4.57	{3} M=5.82	{4} M=4.55	{5} M=3.85	{6} M=4.53
Professional athletes G/G	{1}		0.8999	**0.0036**	0.6243	0.2733	0.6407
Professional athletes A/A	{2}			0.1193	0.9795	0.4811	0.9605
Professional athletes A/G	{3}				**0.0012**	**0.0146**	**0.0024**
Control G/G	{4}					0.3430	0.9457
Control A/A	{5}						0.3670
Control A/G	{6}

M – mean.

Significant statistical impact of being a professional athlete and *DRD2* rs1079597 genotype was demonstrated for the score of the Conscientiousness scale. There was a statistically significant effect of *DRD2* rs1079597 genotype interaction and being a professional athlete or nor (control group) on the Conscientiousness scale (F_2.3389_ = 3.23; *p* = 0.0406; η2 = 0.016). The power observed for this factor was 61%, and approximately 1,6% was explained by the polymorphism of the *DRD2* rs1079597 and being a professional athlete or nor (control group) on Conscientiousness score variance. Table 6 presents the post hoc test results.

**TABLE 6:** Post hoc test (Least Significant Difference) analysis of interactions between the Professional athletes /Control and *rs1079597* and Conscientiousness scale.

rs1079597 and Conscientiousness scale							
		{1} M=7.26	{2} M=7.71	{3} M=6.70	{4} M=5.80	{5} M=4.43	{6} M=6.21
Professional athletes G/G	{1}		0.5837	0.2052	< 0.0001	0.0005	0.0006
Professional athletes A/A	{2}			0.2566	**0.0185**	**0.0035**	0.0698
Professional athletes A/G	{3}				**0.0389**	**0.0109**	0.2922
Control G/G	{4}					0.0925	0.1712
Control A/A	{5}						**0.0329**
Control A/G	{6}

M–mean.

The post hoc test showed that professional athletes with the AG genotype had a higher level of Openness compared to the control group with the GG (*p* = 0.0012), AA (*p* = 0.0146) and AG (*p* = 0.0024) genotypes. Moreover, professional athletes with the GG genotype showed a lower level of Openness compared to professional athletes with the AG genotype (*p* = 0.0036). Table 5 shows the results of the post hoc test.

The post hoc test showed that professional athletes with the GG genotype had higher scores on the Conscientiousness scale compared to the control group with the GG (*p* < 0.0001), AA (*p* = 0.0005) and AG (*p* = 0.0006) genotypes. Professional athletes with the AA genotype obtained higher scores on the Conscientiousness scale compared to the control group with the GG (*p* = 0.0185) and AA (*p* = 0.0035) genotypes. Similarly, professional athletes with the AG genotype showed a higher level on Conscientiousness compared to the control group with the GG (*p* = 0.0389) and AA (*p* = 0.0109) genotypes. Table 6 shows the results of the post hoc test.

## DISCUSSION

One controversial issue is whether talent or long-term experience enhances athletic performance [24]. Progress in sports science has emphasised that athletic performance is influenced by many factors, including physiology and environment [25]. Furthermore, research in recent years has highlighted the possible role of the genetic background of athletes in their performance, leading to the emergence of a new field of science known as sport genetics [26–28]. Research into sports participation has increasingly pointed to inherent biological mechanisms as influential factors alongside psychosocial and environmental elements [29–31]. In the context of sports involvement, both twin studies and family resemblance models suggest a genetic transmission of behavioural tendencies [32–34]. Most family resemblance studies have demonstrated a moderate correlation with the heritability of sports participation (around 0.25), with genetics and environmental factors significantly contributing to sports participation among twins [35].

The study had three specific objectives: (1) to conduct an association analysis of the DRD2 gene Tag1B rs1079597 polymorphism in a group of professional athletes compared to controls; (2) to perform a personality trait analysis, measured by the NEO Five-Factor Inventory, and to compare it between the aforementioned groups; and (3) to conduct an interaction analysis of the measured personality traits, rs1079597 and professional sport participation.

The dopamine D2 receptor gene was chosen for analysis as a strong candidate gene for physical activity behaviour, given its role in locomotor control [36] and reward mechanisms [37–40]. Exercise behaviour may be associated with a rewarding effect. In fact, a feeling of pleasure as a consequence of an exercise bout is thought to be a crucial determinant of exercise participation [41, 42]. Animal studies on brain neurotransmitter physiology provide some evidence for exerciseinduced pleasure. Endurance training in rats has been shown to alter the number of brain dopamine-binding sites [43] and the metabolism of brain dopamine [43, 44]. In humans, increased plasma dopamine levels have been observed during both short and prolonged [45–47] exercise bouts. However, the effect of endurance training on brain dopamine levels in humans is not yet fully understood [48, 49]. Since the *DRD2* gene is implicated in reward mechanisms, some studies have emphasised that exercise addiction is associated with athletic performance. For instance, Cetin et al. [50] have shown that athletes with high exercise addiction gave lower performances independent of the branches. The presented study analysed the association of the *Taq1B* genotypes and alleles in a group of professional athletes and controls. We found statistically significant associations with the GG genotype and G allele being more frequent in the athletes’ group and the AG genotype and A allele in the control group. Michałowska-Sawczyn et al. [51] analysed a number of *DRD2/ANKK1* polymorphic sites, including the *Taq1B*, in a group of martial arts athletes. They obtained similar results, i.e., the GG genotype and the G allele were significantly more frequent in the group of athletes. To the best of the author’s knowledge, there are no other studies on *Taq1B* in relation to professional or non-professional sports participation. However, there are findings regarding other polymorphic sites in the *D2* gene and other dopaminergic genes. In a cross-sectional study, Jozkow et al. [52] found no relationship between sports participation and dopamine receptors D2 and D4 in Polish men. Simonen et al. [48] found a significant association between *DRD2* and sports participation, but only among women. Lee et al. [53] addressed the issue of uncertain causality by using a longitudinal approach to investigate the impact of dopamine receptor genes on sports participation. Although their findings were limited to male students, they shed light on the long-term effects of *DRD2* on sports participation. A study by Świtała et al. [54] analysed the association between a few *DRD2* single nucleotide polymorphisms (SNPs), i.e. rs1076560, rs12364283, rs1799732, rs1800497, and rs1800498, and the body’s response to regular physical activity in a group of females. Performed analysis revealed that individuals with the CC rs1076560 genotype in response to training had a decrease in the basal metabolic rate. Additionally, the haplotypic analysis indicated haplotypes associated with a post-training decrease in glucose level, an increase in the basal metabolic rate and the fat-free mass and a decrease in low-density lipoprotein cholesterol (LDL). Another study by Niewczas et al. [55] regarding *DRD2* rs1799732 in mixed martial arts (MMA) athletes showed no significant results. No significant results were obtained in a study by Chmielowiec et al. [12] regarding martial arts athletes and the same polymorphic site. In a recent study, Bayraktar et al. [56] found no statistically significant differences in allelic and genotypic frequencies of *ANKK1* rs1800497 polymorphism between endurance athletes, sprint/power athletes and controls. Michałowska-Sawczyn et al. [51] found that the number of athletes with the G/G genotype was also higher, although no significant differences were observed in their study. A higher prevalence of the G/G genotype has also been reported in other studies of elite rugby players [57, 58]. The results of the study by Bayraktar et al. [56] found no significant association between the rs1800497 and athletic performance. However, a significant association was found when the duration of an elite athlete’s professional career was taken into account. This suggests that the rs1800497 polymorphism may be used as a marker for predicting the duration of an elite athlete’s professional career.

Athletes are distinguished from amateurs and non-athletes by their extreme physical exertion with a high risk of physical injury, tolerance of emotional stress in social situations, maximisation of efficiency, long-term goals and motivation to perform at high levels while being able to delay gratification [59, 60]. Our analysis of the personality traits revealed that individuals in the group of athletes had significantly higher scores in the assessment of the Extraversion and Conscientiousness scales. Additionally, we performed the interaction analysis revealing a significant impact of being a professional athlete and the *Taq1B* genotype on the Openness and Conscientiousness scales. Of particular interest are the higher results of these traits in professional athletes with the GG and AG genotype. Niewczas et al. [55] analysed MMA athletes’ character and personality traits by means of the Revised Temperament and Character Inventory (TCIR), revealing significantly lower scores on scales of Harm Avoidance and Reward Dependence and higher scores on the Self-directedness scale. Additionally, there was a significant effect of a complex factor of the *DRD2* rs1799732 genotype on Reward dependence in both groups, and the *DRD2* rs1799732 genotype was related to cooperation ability. In a study of martial arts athletes, Chmielowiec et al. [12] obtained similar results, i.e. individuals in the martial arts group obtained significantly higher scores on the Self-directedness scale and lower on the Harm avoidance scale. In this study group, a significant effect of a complex factor of the *DRD2* rs1799732 genotype was found on Reward dependence. The typical profile of athletes in terms of the Big Five personality traits is low in Neuroticism, high in Extraversion and Conscientiousness, with average levels of Openness to experience and Agreeableness [61, 62]. The champions of team sports exhibit lower scores of Neuroticism and higher scores of Extraversion and Openness to experience. The remaining factors did not show any statistical difference from the other players [63–65]. It is important to note that higher-class and successful athletes are less Neurotic and more Extroverted, open-minded, pleasant and Conscientious than the rest of the athletes without notable results [65–67]. The low level of Neuroticism and high levels of other personality traits can benefit athletes, distinguishing champions from other competitors. This has been confirmed in studies of martial artists [68] and individual sports athletes [69]. Piepiora [70] analysed personality profiles of Polish players in senior age from 10 team sports. The results indicate significant personality differences in the following traits: Neuroticism, Extraversion, Agreeableness and Conscientiousness. The largest effect was observed for Neuroticism. Additionally, American football players showed higher levels of Neuroticism compared to rugby and football players. Compared to football, indoor volleyball, and rugby players, Ultimate Frisbee players exhibit significantly lower levels of Extraversion. Additionally, Ultimate Frisbee players demonstrate lower levels of Conscientiousness when compared to basketball, football, beach volleyball, and rugby players. The study also compared champions and other team sports players. The study found significant differences in Neuroticism and Extraversion, while the difference in Openness to experience was not statistically significant after applying the Bonferroni correction. The effect size was very strong for Neuroticism and moderately strong for Extraversion and Openness to experience. The champions of team sports were found to have lower scores of Neuroticism and higher scores of Extraversion and Openness to experience compared to other players [70]. Bäckmand et al. [61] analysed the personality and mood of former athletes in middle and old age. They found no group differences in Extroversion, Neuroticism and life satisfaction, but not in hostility. Sportsmen who practised power/fighting and team sports were more Extroverted than the controls. Shooting and endurance athletes had lower Neuroticism scores than compared to controls. Endurance, power/fighting, team and shooting athletes were more satisfied with their lives than the controls [61].

The study has several limitations that need to be acknowledged. Firstly, the sample size is small, making it difficult to generalise the findings. Secondly, the analysis was limited to athletes (women and men) from various sports disciplines. In the future, the results should be repeated with a larger group of athletes from a more homogeneous range of sporting disciplines. Thirdly, only one polymorphic site was examined, limiting the cumulative assessment of the genetic background.

## CONCLUSIONS

In conclusion, despite its limitations, this study highlights the significant associations between the *DRD2 Taq1B* polymorphic site and personality traits in a group of professional athletes. It also demonstrates the impact of *Taq1B* and professional sportsmanship on personality traits as measured by NEO-FFI. This suggests that genetic factors and professional sportsmanship both play a role in shaping an athlete’s personality traits. Additionally, in the future, the results of studies regarding the personality profile and polymorphic sites could be useful in both the recruitment to professional athletes teams and understanding the athlete’s needs resulting in personalised coaching.
